# Influence of fast-track programs on patient-reported outcomes in total hip and knee replacement (THR/TKR) at Swedish hospitals 2011–2015: an observational study including 51,169 THR and 8,393 TKR operations

**DOI:** 10.1080/17453674.2020.1733375

**Published:** 2020-02-28

**Authors:** Urban Berg, Annette W-Dahl, Ola Rolfson, Emma Nauclér, Martin Sundberg, Anna Nilsdotter

**Affiliations:** a Department of Orthopaedics, Clinical Sciences, Sahlgrenska Academy, Gothenburg University;; b Department of Surgery and Orthopaedics, Kungälv Hospital;; c Department of Orthopedics, Clinical Sciences Lund, Lund University;; d The Swedish Knee Arthroplasty Register;; e Department of Orthopedics Sahlgrenska University Hospital;; f The Swedish Hip Arthroplasty Register, Sweden

## Abstract

Background and purpose — Fast-track care programs have been broadly introduced at Swedish hospitals in elective total hip and knee replacement (THR/TKR). We studied the influence of fast-track programs on patient-reported outcomes (PROs) 1 year after surgery, by exploring outcome measures registered in the Swedish arthroplasty registers.

Patients and methods — Data were obtained from the Swedish Knee and Hip Arthroplasty Registers and included TKR and THR operations 2011–2015 on patients with osteoarthritis. Based on questionnaires concerning the clinical pathway and care programs at Swedish hospitals, the patients were divided in 2 groups depending on whether they had been operated in a fast-track program or not. PROs of the fast-track group were compared with not fast-track using regression analysis. EQ-5D, EQ VAS, Pain VAS, and Satisfaction VAS were analyzed for both THR and TKR operations. The PROMs for TKR also included KOOS.

Results — The differences of EQ-5D, EQ VAS, Pain VAS, and Satisfaction VAS 1 year after surgery were small but all in favor of fast-track for both THR and TKR, also in subscales of KOOS for TKR except KOOS QoL. However, the effect sizes as measured by Cohens’ d formula were < 0.2 for all PROs, in both THR and TKR.

Interpretation — Our results indicate that the fast-track programs may be at least as good as conventional care from the perspective of PROs 1-year postoperatively.

Fast-track care programs in elective total hip and knee replacement (THR and TKR) were introduced in Europe at the beginning of the 2000s (Husted et al. 2006, Pilot et al. 2006). Using evidence-based methods in preparation and perioperative care aims to reduce surgical and psychological stress and accelerate recovery after surgery (Kehlet et al. 2008). The care concept has been spread worldwide (Antrobus and Bryson 2011, Christelis et al. 2015, Stowers et al. 2016), resulting in short perioperative hospital stay, and is considered to be safe and well tolerated by patients (Machin et al. 2013, Zhu et al. 2017, Deng et al. 2019, Wainwright and Kehlet 2019). During the last few years an increasing number of ambulatory arthroplasties have been performed as outpatients with maintained safety and short-term outcome (Goyal et al. 2017), Gromov et al. 2019, Coenders et al. 2020). The patients’ experiences and degree of satisfaction have been explored in qualitative studies (Specht et al. 2016, Strickland et al. 2018) and by self-made questionnaires concerning satisfaction rating of the care (Husted and Holm 2006, Specht et al. 2015, Winther et al. 2015).

Patient reported outcomes (PROs) after THR and TKR with fast-track programs have been reported using both generic and disease-specific questionnaires (Larsen et al. 2010, 2012, Winther et al. 2015). The follow-up periods have been of different length and only a few of them had a control group with standard care (Larsen et al. 2008, Machin et al. 2013). The PROs with fast-track programs have been compared with PROs from an age- and sex-matched population (Larsen et al. 2010, 2012), and the THR patients but not TKR patients reached the population level after 12 months. In a study from Norway the PROs after 12 months were lower than the matched population level but similar to register-based average gain in general health (EQ-5D) in THR patients (Winther et al. 2015). Brock et al. (2017) studied the length of stay and its impact on WOMAC and SF-36 1 year after surgery. They found a slight improvement of SF-36 associated with shorter LOS but no significant influence on WOMAC. The question remains whether PROs 1 year after THR and TKR are better with fast-track or not compared with conventional care programs.

In Sweden, fast-track programs have been broadly implemented for hip and knee replacements during 2011–2015. We studied the influence of the fast-track programs on PROs in elective THR and TKR 1 year after surgery by exploring the PROs registered in the Swedish hip and knee arthroplasty registers (SHAR and SKAR).

## Patients and methods

### Source of data (Figure 1)

Since 2008 all Swedish hospitals performing elective THR have participated in the PROM program of the SHAR with data preoperatively and postoperatively after 1 year. Data completeness with both pre- and postoperative PROMs is around 75%. The SKAR has a PROM project, which started in the Region Skåne, in the south of Sweden, as a pilot project in 2008. From 2013 an increasing number of orthopedic clinics outside the pilot region have joined the project, and in 2015 there were 15 Swedish hospitals performing TKR participating. PROM data are collected preoperatively and 1 year postoperatively with a completeness of more than 70%.

### Definition of cohorts

The study was based on questionnaires concerning the clinical pathway and care program in elective THR and TKR at Swedish hospitals (Kärrholm et al. 2016). The survey aimed to define when a fast-track program had been introduced. The operations at hospitals responding to the questionnaire were divided into 2 groups depending on whether the operations were made in a fast-track program or not. The definition of fast-track was based on the following criteria: (1) admission on the day of surgery; (2) mobilization within 3–6 hours after operation; and (3) functional discharge criteria in practice (Berg et al. 2018). The functional criteria were: ability to get in and out of bed independently, dress, go to the toilet, walk with crutches, and have sufficient pain treatment. The hospitals in the fast-track cohort reported a median LOS of 2–4 days and the hospitals in the not fast-track group 4–7 days.

We got information from 64 of 83 Swedish hospitals performing hip replacements during the period 2011–2015. Operations at hospitals not responding to the questionnaire were used as a 3rd cohort to get an overview of THR on a national level. The cohort with unknown care program for THR consisted of operations at 19 different hospitals, mostly low-volume hospitals. Thus, there were 3 cohorts of THR, 1 with fast-track, 1 defined as not fast-track, and 1 cohort with unknown care program.

For knee replacements the care programs could be defined as fast-track or not fast-track at all 15 hospitals participating in the PROM program based on the questionnaires to the hospitals. Consequently, the TKR operations could be divided into 2 cohorts, fast-track and not fast-track.

### Data collection

Data were obtained from the SKAR and SHAR and included TKR operations (NGB29, NGB39, and NGB49) and THR operations (NFB29, NFB39, NFB49, and NFB62) on patients with osteoarthritis in the knee (M17.0–M17.5) and the hip (M16.0–M16.9) during the period 2011–2015. Every operation was counted even if patients were operated bilaterally. For THRs, PROM data were collected from SHAR using the generic health status measure EQ-5D (EuroQolGroup 1990) with 3 levels of the dimensions mobility, self-care, usual activities, pain/discomfort, and anxiety/depression. In addition, the visual analogue scale (VAS) with a range from 0 to 100 was used for general health, pain, and satisfaction with surgery. For general health (EQ VAS) the score 0 represents the worst and 100 the best. For Pain VAS and Satisfaction VAS the best score is 0 and 100 the worst outcome. Delta values were used to measure improvement by comparing the preoperative values with the values 1 year postoperatively. The satisfaction (VAS) score was categorized into 5 groups: very satisfied (0–20), satisfied (21–40), neither dissatisfied nor satisfied (41–60), dissatisfied (61–80), and very dissatisfied (81–100). For TKRs the same outcome measures were explored, but according to the PROM project in the SKAR it also included the Knee injury and Osteoarthritis Outcome Score (KOOS) (Roos et al. 1998) with the 5 subscales Pain, Other Symptoms, Activity in Daily Life function (ADL), Sport and recreation function (Sport/Rec), and Knee related Quality of Life (QoL). All subscales have a range from 0 to 100 where the highest scores represent the best outcomes.

### Statistics and data analysis

The 3 cohorts (fast-track, not fast-track, and unknown care program) of THR and the 2 cohorts of TKR operations, respectively, were presented with descriptive statistics on demographic and surgical data. The EQ-5D index (Burstrom et al. 2014, Nemes et al. 2015), EQ VAS, Pain VAS, and Satisfaction VAS postoperative scores 1 year after surgery were compared between the not fast-track and the fast-track groups using regression analysis in 3 steps. First a univariable analysis without adjustments was made. In order to reduce bias in the effects of fast track on PRO outcomes after 1 year, 2 multivariable analyses were undertaken: the 1st of the 2 multivariable analyses included adjustment for patient factors such as age, sex, BMI, Charnley category, and the preoperative scores. These factors may influence how patients report their health status 1 year after surgery (Gordon et al. 2013). Finally, the adjustment also included type of fixation and incision in THR. For TKR type of anesthesia, use of tourniquet, and operation time were included. The significance of each covariate was tested (Wald’s test) before being included in the models. The regression coefficients were presented with 95% confidence interval (CI). The effect sizes (standardized mean differences) for the difference between fast-track and not fast-track as measured by the change from pre- to 1-year post-operation in PROs were calculated using Cohen’s d formula (Cohen 1992). Statistical analyses were performed using R version 3.6.1 (R Foundation for Statistical Computing, Vienna, Austria).

### Ethics, funding, and potential conflicts of interest

Ethical approval was given by the Regional Ethical Review Board in Gothenburg (Dnr: Exp. 2019-01-10, 2019-00559/1095-18). No funding and no competing interests were declared.

## Results

### PROs in THR

The demographic variables were similar in the fast-track and not fast-track group. However, the proportion of cemented fixation and posterior approach was higher in the not fast-track group (Table 1). In the cohort with unknown care program representing less than 10% of the THRs in Sweden, there were more males, the mean age was lower, and the proportion of uncemented fixations was higher.

**Table 1.** Demographics and surgical data on THR operations 2011–2015 in patients with osteoarthritis and complete PROM data. Values are n (%) unless otherwise specified

Variable	Not fast-track n = 19,237	Care program Fast-track n = 27,615	Unknown n = 4,317
Age, mean (SD)	69 (10)	68 (10)	66 (10)
Female sex	11,071 (58)	15,537 (56)	2,269 (53)
BMI, mean (SD)	28 (5)	27 (4)	27 (4)
Year of operation			
2011	5,998 (31)	3,373 (12)	1,170 (27)
2012	4,845 (25)	4,571 (17)	1,085 (25)
2013	3,820 (20)	5,793 (21)	796 (18)
2014	2,879 (15)	6,642 (24)	683 (16)
2015	1,695 (9)	7,236 (26)	583 (14)
Charnley class			
A	9,259 (48)	13,885 (50)	2,112 (49)
B	2,330 (12)	3,362 (12)	514 (12)
C	7,648 (40)	10,368 (38)	1,691 (39)
Fixation			
Cemented	13,469 (70)	17,447 (63)	1,811 (42)
Hybrid	313 (2)	998 (4)	73 (2)
Uncemented	3,278 (17)	5,220 (19)	1,551 (36)
Reverse hybrid	2,130 (11)	3,840 (14)	768 (18)
Resurfacing	47 (0)	95 (0)	114 (3)
Incision			
Direct lateral	6,897 (36)	14,643 (53)	1,940 (45)
Posterior	12,015 (63)	12,965 (47)	2,334 (54)
Other	325 (2)	4 (0)	43 (1)

The pre- and postoperative PROs for THRs were similar in the 3 cohorts, with only small differences between the groups (Table 2). Complete data with numbers of operations within each severity level of the EQ-5D-3L are presented in Table 3, see Supplementary data.

**Table 2.** Mean values (SD) and change (Delta (SD)) in PROs in THRs with complete data preoperatively and 1 year postoperatively

	Preoperatively			1 year postoperatively			Delta		
PRO	Not fast-track	Fast-track	Unknown	Not fast-track	Fast-track	Unknown	Not fast-track	Fast-track	Unknown
EQ-5D index	0.74 (0.11)	0.73 (0.11)	0.74 (0.11)	0.88 (0.11)	0.88 (0.11)	0.89 (0.11)	0.14 (0.13)	0.15 (0.13)	0.15 (0.13)
EQ VAS	57 (22)	58 (22)	57 (22)	76 (20)	78 (20)	78 (20)	20 (26)	19 (26)	21 (25)
Pain VAS	63 (15)	63 (15)	66 (16)	14 (18)	13 (17)	12 (18)	–49 (22)	–50 (22)	–54 (22)
Satisfaction VAS				16 (20)	14 (20)	14 (20)			

**Table 4.** Categories of satisfaction with the THR operation. Values are n (%)

Satisfaction (VAS)	Not fast-track	Fast-track	Unknown
Very satisfied (0–20)	13,835 (72)	20,581 (75)	3,303 (77)
Satisfied (21–40)	3,284 (17)	4,207 (15)	595 (14)
Neither dissatisfied nor satisfied (41–60)	1,237 (6.4)	1,643 (5.9)	244 (5.7)
Dissatisfied (61–80)	529 (2.7)	775 (2.8)	97 (2.2)
Very dissatisfied (81–100)	352 (1.8)	409 (1.5)	78 (1.8)

Compared with the not fast-track group a slightly higher proportion of very satisfied patients (VAS 0–20) was seen in the group with fast-track program, but the proportion of patients considered as very satisfied and satisfied (VAS 0–40) was similar in the 3 cohorts, at 89–90% (Table 4).

In the regression analyses without adjustments the deviations from the reference were in favor of fast-track; with adjustments for all variables the deviations were equal or larger. However, the differences were small, < 2 on the scale from 0 to 100 for PROMs assessed by the VAS scale (Figure 2). The effect sizes were < 0.2 for all PROs, indicating small effects of the care program.

**Figure 1.**
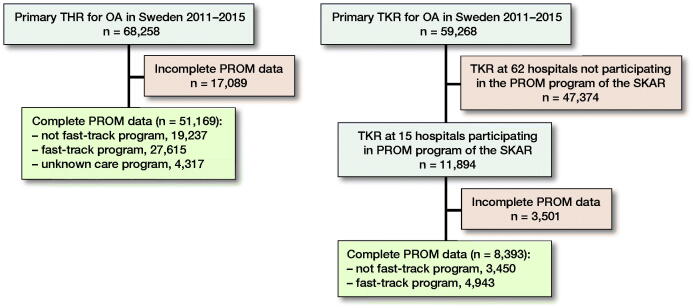
Flow chart of the study. THR = total hip replacement, TKR = total knee replacement, and OA = osteoarthritis.

**Figure 2.**
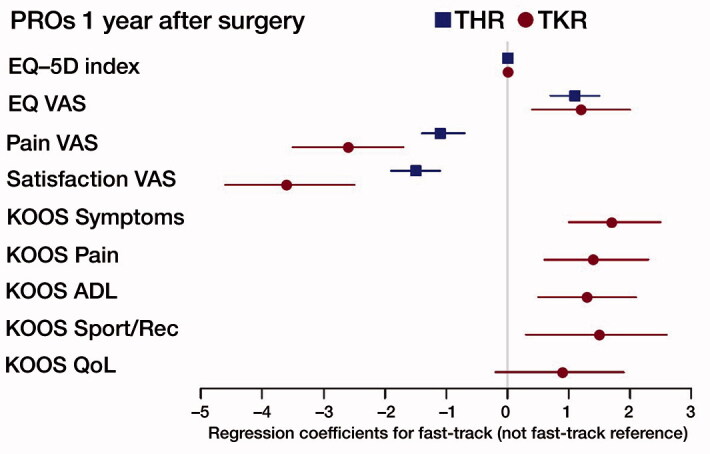
Multivariable regression analysis of PROs 1 year after THR/TKR with 95% CI. Regression coefficients for fast-track (not fast-track reference). Adjustments for age, sex, BMI, Charnley class, preop scores, and year of operation. For THR also adjustment for implant fixation method and surgical approach.

### PROs in TKR

In the fast-track group there was a higher proportion of operations with general anesthesia, tourniquet usage was less common, and the mean operation time was shorter. The demographics of the 2 cohorts were similar, with a slightly higher proportion of males in the fast-track group (Table 5).

**Table 5.** Demographics and surgical data on TKR operations 2011–2015 in patients with osteoarthritis and complete PROM data. Values are n (%) unless otherwise specified

	Care program	
Variable	Not fast-track n = 3,450	Fast-track n = 4,943
Age, mean (SD)	69 (9)	69 (9)
Female	2,047 (59)	2,753 (56)
BMI, mean (SD)	29 (5)	29 (4)
Year of operation		
2011	928 (27)	79 (2)
2012	491 (14)	590 (12)
2013	567 (16)	1,099 (22)
2014	668 (19)	1,503 (30)
2015	796 (23)	1,672 (34)
Charnley class		
A	854 (25)	1,008 (20)
B	1,178 (34)	1,857 (38)
C	1,418 (41)	2,078 (42)
Type of anesthesia		
General	722 (21)	1,610 (33)
Spinal	2,639 (77)	3,272 (66)
Other	83 (2)	51 (1)
Tourniquet	2,302 (67)	2,408 (49)
LIA	3,338 (97)	4,856 (98)
Operation time minutes, mean (SD)	75 (22)	60 (24)

LIA = Local infiltration analgesia with/without catheter

The improvement preoperatively to 1 year postoperatively was considerable regarding pain and health-related quality of life (HRQoL) in both cohorts, but the differences in postoperative mean scores between the cohorts were small, though slightly better in the fast-track group (Table 6). Each of the 5 questions of the EQ-5D is presented in Table 7, see Supplementary data.

**Table 6.** Mean values (SD) and change (Delta (SD)) in PROs in TKRs with complete data preop and 1 year postoperatively

	Preoperatively		1 year postoperatively		Delta	
PRO	Not fast-track	Fast-track	Not fast-track	Fast-track	Not fast-track	Fast-track
EQ5D index	0.78 (0.11)	0.77 (0.11)	0.87 (0.11)	0.88 (0.11)	0.10 (0.12)	0.11 (0.13)
EQ VAS	67 (22)	66 (23)	76 (20)	77 (20)	9 (24)	11 (25)
Pain VAS	63 (18)	64 (17)	20 (20)	17 (20)	–43 (24)	–47 (25)
Satisfaction VAS	21 (23)	17 (23)				

**Table 7.** EQ-5D3L data in TKR patients with complete responses preoperatively and 1 year postoperatively. Values are n (%)

	Preoperatively		1 year postoperatively	
	Not fast-track	Fast-track	Not fast-track	Fast-track
Respons	n = 4,528	n = 7,366	n = 4,528	n = 7,366
PROM responses	3,450 (76)	4,943 (67)	3,450 (76)	4,943 (67)
Mobility				
No problems	468 (14)	581 (12)	2,135 (62)	3,198 (65)
Some problems	2,965 (86)	4,359 (88)	1,309 (38)	1,741 (35)
Extreme problems	17 (0.5)	3 (0.1)	6 (0.2)	4 (0.1)
Self-care				
No problems	3,229 (94)	4,694 (95)	3,277 (95)	4,741 (96)
Some problems	190 (5.5)	210 (4.2)	157 (4.6)	171 (3.5)
Extreme problems	31 (0.9)	39 (0.8)	16 (0.5)	31 (0.6)
Usual activities				
No problems	2,071 (60)	2,653 (54)	2,658 (77)	3,875 (78)
Some problems	1,217 (35)	2,044 (41)	736 (21)	985 (20)
Extreme problems	162 (4.7)	246 (5.0)	56 (1.6)	83 (1.7)
Pain/Discomfort				
No problems	74 (2.1)	78 (1.6)	1,171 (34)	1,847 (37)
Some problems	2,222 (64)	2,964 (60)	2,092 (61)	2,837 (57)
Extreme problems	1,154 (33)	1,901 (39)	187 (5.4)	259 (5.2)
Anxiety/Depression				
No problems	2,278 (66)	3,356 (68)	2,643 (77)	3,961 (80)
Some problems	1,082 (31)	1,492 (30)	728 (21)	913 (19)
Extreme problems	90 (2.6)	95 (1.9)	79 (2.3)	69 (1.4)

The proportions of very satisfied and satisfied (VAS 0–40) were 86% in the fast-track group and 83% in the not fast-track group (Table 8). The difference was larger in the category of very satisfied patients (VAS 0–20), with 72% in the fast-track group compared with 62% in the group with a care program defined as not fast-track.

**Table 8.** Categories of satisfaction with the TKR operation. Values are n (%)

Satisfaction (VAS)	Not fast-track	Fast-track
Very satisfied (0–20)	2,131 (62)	3,570 (72)
Satisfied (21–40)	720 (21)	670 (14))
Neither dissatisfied nor satisfied (41–60)	345 (10)	386 (7.8)
Dissatisfied (61–80)	157 (4.6)	197 (4.0)
Very dissatisfied (81–100)	97 (2.8)	120 (2.4)

**Figure 3.**
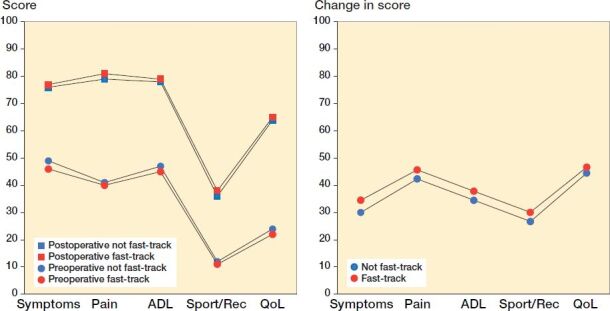
Mean scores (left panel) and improvement (right panel) of the KOOS subscales (0–100) preoperatively and 1-year postoperatively.

Preoperative and postoperative mean scores in the subscales of KOOS for both cohorts are illustrated in Figure 3. The improvement, preoperatively to 1 year postoperatively (Delta values) was considerable in all subscales in both cohorts (Figure 3), and the adjusted regression estimate of the effect of care process was in favor of fast-track in all subscales except for the subscale KOOS QoL. The differences between the groups were small (1–2 points) both pre- and 1 year postoperatively. A table with mean scores of the KOOS subscales is available in the Supplementary data (Table 9).

The multivariable regression analysis points to a favorable influence of fast-track in all subscales and outcome measures compared with not fast-track in TKR, but the differences were small (Figure 2). The effect sizes were < 0.2 as for THR. In the Supplementary data a table with adjustments for all procedure-specific variables is available (Table 10).

## Discussion

We found that the fast-track cohort had slightly better PROs in both THR and TKR patients concerning pain, satisfaction, and all dimensions of health outcome. The KOOS scores were statistically significantly better in the fast-track group of TKR patients in all subscales except for the subscale QoL (Figure 2). However, the differences were small, and the clinical relevance may be questioned.

In the regression analysis with adjustments the year of operation had a limited impact on the outcome, and the results in THR and TKR were more influenced by Charnley class C and preoperative score than the care program (Tables 10 and 11, see Supplementary data).

In all cohorts of THR and TKR there were patients operated bilaterally during the period 2011–2015; most of them had a 2-stage operation. Consequently, the outcomes of two operations were recorded in the same patient, but as there are large similarities between unilateral and second 2-stage bilateral THR (Bülow, Nemes and Rolfson, personal communication 2019) we consider that bilaterality does not have a practical influence on the analysis of PROM data in our study.

Previous studies aiming to evaluate PROs after THR or TKR in fast-track programs by using generic and disease-specific questionnaires had shorter follow-up periods (Larsen et al. 2008, Machin et al. 2013) or did not have a control group with standard care (Larsen et al. 2010, 2012, Winther et al. 2015). In our study the Swedish value set was used to calculate the EQ-5D index (Burstrom et al. 2014), which is not the same as the value sets used in other countries. Consequently, it is difficult to compare our results with previous publications from hospitals outside Sweden. In our study disease-specific questionnaires were not used for hips, but Larsen et al. (2010) found that generic and disease-specific outcomes such as EQ-5D and Harris Hip Score were strongly associated in THR.

In THR it has been reported that uncemented fixation is associated with better PROs 1 year after THR (Rolfson et al. 2016) and in the fast-track cohort of our study there were in fact more uncemented and hybrid fixations. Posterior approach is also associated with better PROs compared with direct lateral approach (Lindgren et al. 2014), but the fast-track group had contrarily a higher proportion of direct lateral approach. This may explain why the difference in favor of fast-track was slightly larger after adjustment for surgical approach.

In the fast-track cohort of TKR the use of a tourniquet was less common, but the influence of a tourniquet on functional outcome is inconclusive according to previous studies (Ledin et al. 2012, Zhang et al. 2014, Harsten et al. 2015, Zhou et al. 2017). Ledin et al. (2012) showed in a small randomized study an improved range of motion (ROM) persisting after 2 years and Harsten et al. (2015) showed no effect regarding postoperative pain and muscle strength when a tourniquet was not used while Zhang et al. (2014) and Zhou et al. (2017) found a better ROM in early stage after surgery when not using a tourniquet.

We have no evidence from the literature or from our findings that variables related to the surgical intervention in TKR may have an influence on the PROMs 1 year after surgery. Thus, in our regression analysis we have preferred to present the results with adjustment for the demographic variables, preop scores, and year of operation but not for the surgical variables.

The interpretation of results should always be made with caution. In a multinational evaluation of minimum clinical important improvement (MCII) of generic outcomes with scores from 0 to 100 used in rheumatic diseases including hip and knee osteoarthritis, the conclusion was that an absolute improvement of 15 out of 100 or 20% relative improvement was considered as MCII (Tubach et al. 2012). The MCII differs between diagnoses and whether the scores are in the upper or lower range. It has also been suggested that a change of 8 points on the KOOS subscale score ranging from 0 to 100 could be the minimal perceptible clinical improvement (MPCI) (Roos and Lohmander 2003).

However, if the differences are too small to be considered as clinically relevant in an individual, it does not mean that the improvement on group level is without importance. Hip and knee replacements are powerful interventions with a high effect size, especially regarding pain and physical function (Jones and Pohar 2012) and we cannot expect more than a small additional improvement of outcome when the care programs are optimized. The responsiveness and ceiling effects of the PROs are also factors to consider in detecting small changes (Greene et al. 2015). Further, outcome may also differ depending on the PROM used (W-Dahl et al. 2014).

It is difficult to define specific factors or procedures in the fast-track clinical pathway with the strongest positive impact on PROs 1 year postoperatively. Preoperative information from a multi-professional team and clearly communicated functional discharge criteria may reduce the anxiety and mental stress. The logistic frame aiming for a short LOS stimulates all professionals to contribute in preoperative preparation as well as coaching during the hospital stay and postoperatively to achieve the goal of rapid recovery.

### Strengths and limitations

Our study explores the influence of fast-track programs in elective THR and TKR in Sweden during a period of nationwide implementation with register data for 5 years. All Swedish hospitals performing hip replacements are included in the PROM program of the SHAR, and this study gives an overview of the influence of fast-track programs on PROs in elective THR. In TKR 15 hospitals of 77 performing knee replacements participated in the PROM project during this period; however, according to register data the demographics and surgical data in the studied group were similar to TKRs in hospitals not participating. The data completeness of the PROM programs was more than 70% for both THR and TKA.

A limitation of the study is that bias can persist, due to imbalance in potential confounding factors outside the care program, which may affect the results. The influence on PROs 1 year postoperatively is multifactorial and variables such as socioeconomic factors and mental health have not been explored.

Fast-track lacks an internationally accepted clear definition. We have used a definition mainly based on logistical criteria in the care of patients undergoing THR and TKR (Berg et al. 2018), but in the fast-track philosophy there are also care principles that are not applied in exactly the same way in all hospitals, for example the use of anti-thrombotic medication. The absence of exact data on LOS is a weakness, but the reported median LOS from the hospitals indicates that fast-track hospitals had a shorter LOS (2–4 days) than the not-fast-track hospitals (4–7 days).

## Conclusion

Fast-track care programs in hip and knee replacements broadly introduced at Swedish hospitals during the period 2011–2015 are associated with slightly better PROs 1 year after the operation compared with programs defined as not fast-track. The small differences may not be of clinical relevance, but our results indicate that fast-track programs may be at least as good as the conventional care from the perspective of PROs. Further studies are needed to identify which factors are the most important in order to refine and further improve the clinical pathway and care process based on the principles of fast-track.

## Supplementary Material

Supplemental Material

## Supplementary data

Tables 3, 7, and 9–11 are available as supplementary data in the online version of this article, http://dx.doi.org/10.1080/17453674.2020.1733375
