# Supplementary material for: Influence of fast-track programs on patient-reported outcomes in total hip and knee replacement (THR/TKR) at Swedish hospitals 2011–2015: an observational study including 51,169 THR and 8,393 TKR operations
Source: Acta Orthop. 2020 Feb 28;91(3):306–12. doi: 10.1080/17453674.2020.1733375 (PMC8023888; doi:10.1080/17453674.2020.1733375)
Supplement: Supplemental Material [file IORT_A_1733375_SM7102.pdf]

Supplementary data

Table 3. EQ-5D-3L data in THR patients with complete responses preoperatively and 1 year postoperatively. Values are n (%)

Respons	Preoperatively<br>Not fast-track<br>n = 25,585	Fast-track<br>n = 35,986	Unknown<br>n = 6,688	1 year postoperatively<br>Not fast-track<br>n = 25,585	Fast-track<br>n = 35,986	Unknown<br>n = 6,688
PROM responses	19,237 (75)	27,615 (77)	4,317 (65)	19,237 (75)	27,615 (77)	4,317 (65)
Mobility						
No problems	1,569 (8.2)	2,296 (8.3)	376 (8.7)	11,682 (61)	17,066 (62)	2,804 (65)
Some problems	17,625 (92)	25,252 (91)	3,928 (91)	7,530 (39)	10,520 (38)	1,506 (35)
Extreme problems	43 (0.2)	67 (0.2)	13 (0.3)	25 (0.1)	29 (0.1)	7 (0.2)
Self-care						
No problems	15,047 (78)	21,550 (78)	3,408 (79)	17,804 (93)	25,920 (94)	4,031 (93)
Some problems	4,006 (21)	5,849 (21)	882 (20)	1,336 (6.9)	1,574 (5.7)	263 (6.1)
Extreme problems	184 (1.0)	216 (0.8)	27 (0.6)	97 (0.5)	121 (0.4)	23 (0.5)
Usual activities						
No problems	7,730 (40)	11,088 (40)	1,654 (38)	14,891 (77)	21,960 (80)	3,424 (79)
Some problems	9,707 (51)	14,010 (51)	2,273 (53)	3,965 (21)	5,188 (19)	826 (19)
Extreme problems	1,800 (9.4)	2,517 (9.1)	390 (9.0)	381 (2.0)	467 (1.7)	67 (1.6)
Pain/Discomfort						
No problems	279 (1.5)	397 (1.4)	71 (1.6)	8564 (45)	12752 (46)	2172 (50)
Some problems	11,093 (58)	15,839 (57)	2,538 (59)	9,750 (51)	13,677 (50)	1,948 (45)
Extreme problems	7,865 (41)	11,379 (41)	1,708 (40)	923 (4.8)	1,186 (4.3)	197 (4.6)
Anxiety/Depression						
No problems	11,466 (60)	16,412 (59)	2,747 (64)	15,039 (78)	22,022 (80)	3,506 (81)
Some problems	7,197 (37)	10,316 (37)	1,444 (33)	3,903 (20)	5,227 (19)	747 (17)
Extreme problems	574 (3.0)	887 (3.2)	126 (2.9)	295 (1.5)	366 (1.3)	64 (1.5)

7

Table 7. EQ-5D3L data in TKR patients with complete responses preoperatively and 1 year postoperatively. Values are n (%)

Respons	Preoperatively<br>Not fast-track<br>n = 4,528	Fast-track<br>n = 7,366	1 year postoperatively<br>Not fast-track<br>n = 4,528	Fast-track<br>n = 7,366
PROM responses	3,450 (76)	4,943 (67)	3,450 (76)	4,943 (67)
Mobility				
No problems	468 (14)	581 (12)	2,135 (62)	3,198 (65)
Some problems	2,965 (86)	4,359 (88)	1,309 (38)	1,741 (35)
Extreme problems	17 (0.5)	3 (0.1)	6 (0.2)	4 (0.1)
Self-care				
No problems	3,229 (94)	4,694 (95)	3,277 (95)	4,741 (96)
Some problems	190 (5.5)	210 (4.2)	157 (4.6)	171 (3.5)
Extreme problems	31 (0.9)	39 (0.8)	16 (0.5)	31 (0.6)
Usual activities				
No problems	2,071 (60)	2,653 (54)	2,658 (77)	3,875 (78)
Some problems	1,217 (35)	2,044 (41)	736 (21)	985 (20)
Extreme problems	162 (4.7)	246 (5.0)	56 (1.6)	83 (1.7)
Pain/Discomfort				
No problems	74 (2.1)	78 (1.6)	1,171 (34)	1,847 (37)
Some problems	2,222 (64)	2,964 (60)	2,092 (61)	2,837 (57)
Extreme problems	1,154 (33)	1,901 (39)	187 (5.4)	259 (5.2)
Anxiety/Depression				
No problems	2,278 (66)	3,356 (68)	2,643 (77)	3,961 (80)
Some problems	1,082 (31)	1,492 (30)	728 (21)	913 (19)
Extreme problems	90 (2.6)	95 (1.9)	79 (2.3)	69 (1.4)

Table 9. Mean values (SD) and change (Delta (SD)) in KOOS data in TKR patients with complete responses preoperatively and 1 year postoperatively

	Preoperatively		1 year postoperatively		Delta	
	Not fast-track n = 4,528	Fast-track n = 7,366	Not fast-track n = 4,528	Fast-track n = 7,366	Not fast-track	Fast-track
Responses	3,450 (76)	4,652 (63)	3,450 (76)	4,652 (63)		
KOOS subscale						
Symptoms	49 (18)	46 (18)	76 (17)	77 (17)	27 (21)	31 (21)
Pain	41 (15)	40 (15)	79 (19)	81 (19)	38 (21)	41 (21)
Daily activity	47 (16)	45 (16)	78 (19)	79 (19)	31 (20)	34 (20)
Sports/rec.	12 (14)	11 (14)	36 (27)	38 (27)	24 (27)	27 (27)
QoL	24 (14)	22 (14)	64 (24)	65 (24)	40 (25)	42 (25)

Table 10. Multivariable regression analysis of EQ5D Index 1 year after TKR with 95% confidence interval (CI) with adjustments for Pre EQ index, year of operation, demographic and procedure-specific variables

	Estimate	(CI)
Not fast-track	ref.	
Fast-track	0.010	(0.005 to 0.014)
Pre EQ index	0.292	(0.271 to 0.312)
Age	0.000	(0.000 to 0.000)
Female sex	–0.004	(–0.009 to 0.000)
BMI	–0.002	(–0.002 to –0.001)
Charnley B	–0.007	(–0.012 to –0.001)
Charnley C	–0.048	(–0.054 to –0.042)
Year of operation	0.002	(0.000 to 0.004)
Anesthesia		
general	–0.009	(–0.014 to –0.004)
other	0.030	(0.013 to 0.047)
No tourniquet	0.002	(–0.002 to 0.007)
Operation time		
30–60 min	–0.002	(–0.007 to 0.003)
>90 min	–0.007	(–0.014 to –0.001)

Table 11. Multivariable regression analysis of EQ5D Index 1 year after THR with 95% confidence interval (CI) with adjustments for age, sex, BMI, Charnley class, preop EQ VAS, year of operation, implant fixation method and surgical approach

	Estimate	(CI)
Not fast-track	ref.	
Fast-track	0.006	(0.004 to 0.008)
Unknown	0.004	(0.001 to 0.008)
Pre EQ Index	0.238	(0.230 to 0.246)
Age	–0.001	(–0.001 to –0.001)
Female sex	–0.011	(–0.013 to –0.010)
BMI	–0.002	(–0.002 to –0.002)
Charnley B	–0.025	(–0.027 to –0.022)
Charnley C	–0.051	(–0.053 to –0.049)
Year of operation	0.000	(0.000 to 0.001)
Hybrid	0.004	(–0.002 to 0.009)
Uncemented	0.005	(0.002 to 0.007)
Reversed hybrid	0.002	(0.000 to 0.005)
Resurfacing	0.006	(–0.007 to 0.018)
Incision other	0.009	(–0.002 to 0.019)
Incision posterior	0.015	(0.013 to 0.017)
